# Uncovering a connection between physiological stress and therapeutic resistance in tumor cells

**DOI:** 10.1186/2051-1426-1-S1-P186

**Published:** 2013-11-07

**Authors:** Jason W Eng, Chelsey Reed, Kathleen M Kokolus, Rose Pitoniak, Bonnie L Hylander, Elizabeth A Repasky

**Affiliations:** 1 Department of Immunology, Roswell Park Cancer Institute, Buffalo, NY, USA

## 

Environmental stress, including that caused by ambient temperature, significantly impacts many biological processes. Under conditions of cold stress, the sympathetic nervous system and a subpopulation of alternatively activated macrophages produce norepinephrine to maintain homeostasis by increasing heat production (thermogenesis). Yet in spite of these dramatic physiological effects, the impact of cold stress has been underappreciated, particularly in pre-clinical mouse models which are used extensively in basic biological research and therapy development. Namely, all animal facilities are maintained between 20-26°C despite the thermoneutral temperature for mice being approximately 30°C. Therefore, mice must expend a great deal of energy to maintain body temperature at 37°C. The full implications of this thermal stress on mouse models of disease, particularly of cancer, are unknown. In preliminary experiments, we compared the growth of tumors and their response to therapies in mice housed at 22°C and 30°C. Although growth rates of human xenografts in SCID mice, a model commonly used for therapy development, were similar in these two conditions, we observed that some tumors responded differently to the same therapies. We found that MiaPaca2 and BxPC-3 pancreatic tumor xenografts showed increased sensitivity to Apo2L/TRAIL and cisplatin when mice were placed at 30°C vs. 22°C. Additionally, we saw a significant decrease in markers of M2 macrophages, a major source of norepinephrine, in the tumors of mice housed at 30°C compared with those at 22°C. Thus, we hypothesize that tumor cells respond to the elevated levels of norepinephrine in cold stressed animals by increasing the expression of key survival molecules. Subsequent analysis of tumors from mice housed at 22°C revealed that the expression of anti-apoptotic molecules Bcl-2, Bcl-xL, and Mcl-1 were elevated compared to those housed at 30°C. Furthermore, our in vitro studies show that norepinephrine can induce the expression of Bcl-2 and Bcl-xL in a time dependent manner human pancreatic tumor cell lines, and that this increased protein expression results in increased resistance to both chemotherapy and death receptor mediated apoptosis. In addition, treatment of tumor-bearing mice with the β-blocker, propranolol, increased the response of tumors in mice housed at 22°C to Apo2L/TRAIL, but not mice housed at 30°C. These results support the strong possibility that the therapeutic response studied in mouse models is significantly impacted by cold stress. This study has important implications both for the development of novel therapies as well as the understanding of how temperature related stress and the activation of thermogenesis could affect drug efficacy in patients.

